# Use of Insulin Degludec/Insulin Aspart in the Management of Diabetes Mellitus: Expert Panel Recommendations on Appropriate Practice Patterns

**DOI:** 10.3389/fendo.2021.616514

**Published:** 2021-03-12

**Authors:** Tevfik Demir, Serap Turan, Kursad Unluhizarci, Oya Topaloglu, Tufan Tukek, Dilek Gogas Yavuz

**Affiliations:** ^1^ Department of Endocrinology and Metabolism, Dokuz Eylul University Faculty of Medicine, Izmir, Turkey; ^2^ Department Pediatric Endocrinology, Marmara University Faculty of Medicine, Istanbul, Turkey; ^3^ Department of Endocrinology and Metabolism, Erciyes University Faculty of Medicine, Kayseri, Turkey; ^4^ Department of Endocrinology and Metabolism, Ankara Yildirim Beyazit University Faculty of Medicine, Ankara City Hospital, Ankara, Turkey; ^5^ Department of Endocrinology and Metabolism, Istanbul University Istanbul Faculty of Medicine, Istanbul, Turkey; ^6^ Department of Endocrinology and Metabolism, Marmara University Faculty of Medicine, Istanbul, Turkey

**Keywords:** expert panel, type 2 diabetes, type 1 diabetes, glycemic control, hypoglycemia, Turkey, IDegAsp, treatment switching

## Abstract

Insulin degludec/insulin aspart (IDegAsp) is a fixed-ratio co-formulation of insulin degludec (IDeg), which provides long-lasting basal insulin coverage, and insulin aspart (IAsp), which targets post-prandial glucose. This expert panel aimed to provide a practical and implementable guidance document to assist clinicians in prescribing IDegAsp in the diabetes management with respect to different patient populations including children and adults with type 1 diabetes (T1D) or type 2 diabetes (T2D) as well as pregnant, elderly and hospitalized patients and varying practice patterns (insulin-naive, insulin-treated, switching from basal, basal bolus and premix regimens). The experts recommended that IDegAsp can be used in insulin-naive T2D patients with poor glycemic control (HbA1c >8.5%) despite optimal oral antidiabetic drugs (OADs) as well as in insulin-treated T2D patients by switching from basal insulin, basal-bolus therapy or premixed insulins in relation to lower risk of nocturnal hypoglycemia, fewer injections and lower intraday glycemic variability, respectively. The experts considered the use of IDegAsp in children with T2D as a basal bolus alternative rather than as an alternative to basal insulin after metformin failure, use of IDegAsp in adult T1D patients as a simplified basal bolus regimen with lesser nocturnal hypoglycemia, fewer injections and better fasting plasma glucose control and in children with T1D as an alternative insulin regimen with fewer injection to increase treatment adherence. The proposed expert opinion provides practical information on use of IDegAsp in different patient populations and practice patterns to assist clinicians, which seems to compensate the need for easily implementable guidance on this novel insulin regimen.

## Introduction

In accordance with worldwide trends for rapidly increasing prevalence of diabetes, it is estimated that 700 million people will be affected by diabetes 2045 (1). A 90% increase was noted in type 2 diabetes (T2D) prevalence in Turkey within the last two decades from 7.2% in 1997–1998 (2) to 13.7% (over 6.5 million people) in 12 years (3). The 2019 data from International Diabetes Federation (IDF) Atlas also revealed a 12.0% (6.6 million people) prevalence of adult diabetics in Turkey, affecting one out of every eight adults, and 25,953 patients with Type 1 diabetes (T1D) (1). Turkey is estimated to be amongst the top 10 countries for number of people with diabetes (20–79 years) by 2045 (1).

Given the association of prolonged glucose load with increased risk of diabetes-related complications and mortality (1, 4), effective early glycemic control is considered critical to achieve sustained and long-term reductions in diabetes-related complications and thereby to reduce mortality and cost of diabetes care related to T1D or T2D (5–7).

Insulin resistance and progressive deterioration of β-cell function in T2D eventually leads to failure to achieve glycemic control via oral antidiabetic drugs (OADs), necessitating insulin initiation (8–10). For having a higher efficacy in HbA1c lowering (1.5–2.5 vs. 0.5–2.0%) than other antidiabetics, insulin therapy is considered effective not only in improving glycemic control but also in reducing the glucotoxic effects and slowing the disease progression (8, 11–13).

However, as the disease progresses patients receiving basal insulin need further intensification of treatment with additional mealtime insulin, either through the addition of mealtime short-acting insulin or by switching to a premixed insulin formulation (8, 14–16). Basal-bolus regimens relatively offer both physiological basal and mealtime insulin responses, but add complexity of three to five separate injections (16–18). Thus, many patients are reluctant to start or adhere with a basal-bolus insulin regimen due to its complexity to administer and titrate, the need for multiple daily injections and a fear of hypoglycemia (18–22).

Premixed insulin formulations contain a fixed proportion of protaminated and non-protaminated (soluble) insulin in a single injection and thus provide basal and mealtime coverage in one injection (23, 24). Notably, data from DROPOUT studies (n = 433 and n = 1,456) (25, 26) in insulin-naive type 2 diabetes patients in Turkey revealed association of premixed insulin treatment with better patient compliance compared to basal-bolus treatment in terms of dose-skipping (19 vs. 52%) and not using insulin for more than a day (22.7 and 61.3%) (25), while in terms of higher treatment adherence (75 vs. 62.8%) compared to basal insulin (26).

Due to some treatment limitations of premixed insulin formulations/options (i.e. inability to adjust the long- and short-acting components separately or adequately treat post-lunch and early-morning hyperglycemia) (24), fixed-ratio co-formulation insulin-based products have been developed that combine a basal and a rapid-acting insulin in a formulation where the two components act independently allowing a simpler insulin regimen with fewer injections alongside basal and prandial insulin coverage (23, 27, 28). Insulin degludec/insulin aspart (IDegAsp) is the first fixed-ratio co-formulation of two different insulin analogues comprising rapid-acting insulin aspart (IAsp) (30%) and ultra-long acting insulin degludec (IDeg) (70%) and considered a simpler insulin regimen non-inferior to premixed formulations and an alternative to basal-only and basal-bolus therapy (23, 28–30).

Although clinical evidence and meta-analysis data support the use of IDegAsp in a wide variety of patient populations for either insulin initiation or intensification (31–40), there is limited guidance on practical clinical use of IDegAsp (41). Available guidance on the use of IDegAsp is limited in terms of addressing common challenges in clinical use (42–44), while American Diabetes Association/European Association for the Study of Diabetes (EASD/ADA) 2018 treatment recommendations may not be applicable in all patient populations (45). Hence, there is a need for guidance document to assist clinicians in using this novel insulin regimen with unique pharmacological properties in real-life clinical practice in terms of dose timing relative to meal(s), daily dosage, treatment intensification or switching from previous antidiabetic therapy as well as different patient populations (adult, children, T1D, T2D, pregnant, elderly, hospitalized).

The proposed expert opinion was therefore prepared by a panel of expert endocrinologists from Turkey to provide a practical and implementable guidance document to assist clinicians for appropriate use of IDegAsp.

## Methods

The present expert panel of endocrinology specialists met to develop an expert opinion and recommendations on appropriate use of IDegAsp in insulin-naïve or insulin-treated diabetic patients including children, adults and special (i.e. elderly, hospitalized) patient sub-groups. The expert panel members were professors, also international speakers and national influencers, with at least 15 years of experience in diabetes management and were from different geographical regions of Turkey. All experts were informed about the study via e-mail by the sponsor and then participated in the consecutive meetings supported by the sponsor to achieve the proposed opinion. The panel critically analyzed recommendations from international guidelines, systemic reviews and meta-analyses, results of randomized control trials focusing on the efficacy and safety of IDegAsp, and agreed on a series of statements supported by scientific evidence and expert clinical opinion to assist clinicians in endocrinology practice. The proposed expert opinion planned to provide a practical and implementable guidance document addressing the appropriate use of IDegAsp in the management of diabetes in terms of (a) IDegAsp-based freedom and flexibility in diabetes care, b) the main meal concept c) use of IDegAsp in pediatric and adult T2D (insulin-naïve, switching from basal insulin, switching from premix insulins) patients, d) use of IDegAsp in T1D patients, e) use of IDegAsp in pregnancy and f) use of IDegAsp in special patient groups (elderly and hospitalized)

### Freedom and Flexibility in Diabetes Care

IDegAsp is the first soluble combination of two different insulin analogues (70 % IDeg and 30 % IAsp), providing IDeg-mediated long and steady basal glucose-lowering effect and IAsp-mediated mealtime glycemic control in a single pen (46). A highly stable structure formed by assembly of di-hexamers held together by side chain-zinc contacts is unique to IDeg (46). At high zinc concentrations, there is no association between IDeg and IAsp monomers, either in the formulation or the injection depot (46). This leads to a product with available insulin components as separate and stable soluble forms in the formulation, thereby avoiding the need for resuspension prior to injection as well as distinct and clearly separated prandial and basal glucose-lowering effects at steady state (23, 29, 46, 47).

IDegAsp works as both premix and basal plus regimens, and can be used as a component of basal bolus regimens as well, since it enables to provide a safe and well-tolerated fasting as well as prandial blood glucose control (28, 42, 43, 48). In addition, the ultra-long duration of action of the basal component of IDegAsp offers the potential for flexible dosing times (29). Thus, either once- or twice daily injection is possible, with IDegAsp dosed with meal(s) having the largest glycemic impact, while it can also be used as part of basal bolus regimen (one IDegAsp and two IAsp doses) (29, 48).

**Figure 1:**
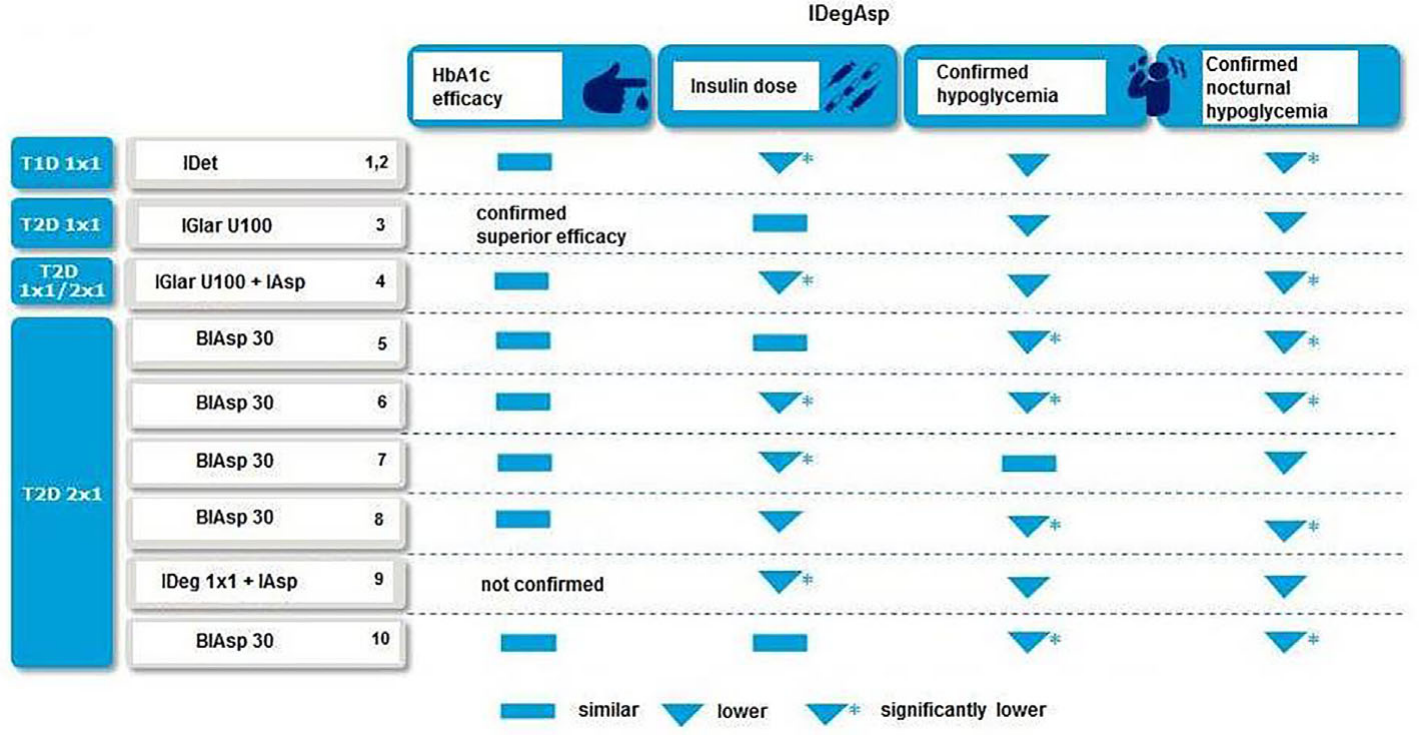
IDegAsp vs. other insulin regimens in clinical trials 1(31); 2(32); 3(33); 4(52); 5(34); 6(35), 7(36); 8(53); 9(38); 10(39).

IDegAsp allows freedom and flexibility in diabetes care with co-formulating basal and bolus insulins in a single injection that allow a simple regimen with fewer injections (43, 48). The efficacy and safety of varying the daily injection time of IDeg has been studied in T2D patients, indicating flexibility of IDeg with achievement of glycemic control (HbA1c improved by 1.28, 1.07, and 1.26% points in IDeg once daily (OD) Flex, IDeg OD, and IGlar OD, respectively, estimated treatment difference [ETD] IDeg OD Flex - IGlar OD: 0.04% points [-0.12 to 0.20], confirming non-inferiority) without a significant increase in the risk of overall confirmed hypoglycemia (estimated rate ratio [RR] IDeg OD Flex/IGlar OD: 1.03 [0.75–1.40], p = NS) and nocturnal confirmed hypoglycemia (estimated RR: 0.77 [0.44–1.35], p = NS) for 26 weeks in patients treated based on 8–40-h intervals between doses (49). Greater flexibility in the day-to-day timing of basal insulin administration may facilitate insulin management for patients, especially for those who consider injecting insulin at the same time each day to be challenging (50). In particular, this could include individuals who travel regularly, while shift workers may also greatly benefit from the freedom to change their dosing schedule (51). Notably, clinical evidence from randomized trials indicated association of IDegAsp with similar and or better HbA1c lowering efficacy, lower insulin dose and lower risk of confirmed hypoglycemia and nocturnal hypoglycemia as compared with basal and basal-bolus regimens, supporting the use of IDegAsp in a wide variety of patient populations for either insulin initiation or intensification (31–39) (**Figure 1**).

In a recent meta-analysis of 5 phase III randomized, 26-week, open-label, treat-to-target trials comparing IDegAsp twice daily (n = 1111) with one of two comparators: premixed insulin (biphasic insulin aspart 30 [BIAsp 30]) twice daily (n = 561) or IDeg once daily + IAsp (n = 136); authors noted similar HbA1c results, significantly lower fasting plasma glucose (FPG) level, lower insulin dose and lower rates of confirmed daily and nocturnal hypoglycemia with IDegAsp vs comparators in all baseline characteristic (40). The authors concluded that IDegAsp retains a consistent safety and efficacy profile in patients with different baseline characteristics (40).

### Main Meal Concept

IDegAsp is administered with the main meal(s) of the day, which is the largest carbohydrate-content meal(s) in adult T2D patients or meal(s) with constant carbohydrate content in child diabetics under multi-insulin therapy (29, 54–56).

While it is of utmost importance to inject IDegAsp with the main meal, it should also be noted that the flexibility in dose timing of IDegAsp allows the main meal to be at any time during the day or at different times from day to day (29, 41).

The expert panel recommends that IDegAsp can be injected before any meal of the day that is rich in carbohydrate, while if a dose is missed, the missed dose should be taken with the next main meal of that day as followed by the usual dosing schedule (29). The expert opinion regarding the recognition of the main meal is to include, besides patient’s dietary anamnesis, an analysis of self-measured blood glucose levels in clinical assessment, as an ideal way to ensure the most carbohydrate-rich meal of the day (**Table 1**).

**Table 1:** Expert Panel Recommendation 1: Main meal concept.

• The largest carbohydrate-content meal(s) at the discretion of the patient • Flexibility in dose timing of IDegAsp allows the main meal to be at any time during the day or at different times from day to day • Alternatively, the main meal can be decided based on analysis of self-measured blood glucose levels

### Use In Pediatric and Adult T2D Patient Populations

#### IDegAsp Therapy in Adult Patients With T2D

##### Initiating in Insulin-Naïve Patients

The utility of IDegAsp in insulin-naïve patients is based on its association with an improved post-prandial glucose (provided by IAsp component) and a stable glucose-lowering effect (provided by IDeg basal component) with lesser 24-h variability when compared to other basal insulins (57). These effects have been demonstrated in clinical trials of insulin-naïve T2D patients treated with IDegAsp versus insulin glargine 100 units/mL (IGlar U100) (33, 55).

In a phase 3, 26-week, open-label, treat-to-target trial in insulin-naïve adults with T2D randomized to once-daily injections of IDegAsp (n = 147) or insulin glargine (IGlar) (n = 149), both ±≤2 OADs, IDegAsp was reported to be associated with superior long-term glycemic control than IGlar (HbA1c: 7.0 vs. 7.3%; HbA1c <7: 43.0 vs. 25%, ETD IDegAsp-IGlar: -0.28% points [-0.46; -0.10](95% CI), p<0.01), with similar FPG (5.7 vs. 5.6 mmol/l; ETD IDegAsp-IGlar: 0.15 mmol/l [-0.29; 0.60](95% CI), p = NS) and insulin doses (both: 0.41 U/kg) and numerically lower rates of overall (by 27.0%, estimated RR IDegAsp/IGlar: 0.73 [0.50; 1.08](95% CI), p = NS) and nocturnal (by 25.0%, estimated RR IDegAsp/IGlar: 0.75 [0.34; 1.64](95% CI), p = NS) hypoglycemia (33). The authors emphasized the superiority of once-daily IDegAsp to IGlar in improving glycemic control and in controlling postprandial glucose excursions without compromising FPG control or safety and with lower rates of overall and nocturnal hypoglycemia (33). Indeed, IDeg itself was previously reported to enable risk reduction for nocturnal hypoglycaemia as compared with IGlar, by providing a very flat glucose-lowering PK/PD profile with low glycemic variability (58, 59). These advantages of IDegAsp seem notable given the association of simpler treatment regimens with better patient compliance and thus higher likelihood of being preferred by physicians as an initiation regimen (60, 61).

Shimoda et al. investigated the efficacy and safety of once daily IDegAsp versus once daily insulin degludec or insulin glargine U300 in insulin-naïve patients on oral hypoglycemic agents for 12 weeks. Subjects were randomized to IDegAsp (n = 26) or basal insulin (n = 26). Percent change in HbA1c, daily insulin doses and frequency of overall hypoglycemia was not significantly different between the groups. Post-hoc analyses revealed that in subjects with HbA1c level less than 8.5%, percent change in HbA1c at week 12 was more pronounced in IDegAsp group while basal insulin was more effective than IDegAsp in subjects with A1c more than 8.5%. The results of this study suggest that the baseline HbA1c level may provide information for initiating IDegAsp or basal insulin in patients with inadequately controlled with oral hypoglycemic agents (62).

The expert panel recommends that in accordance with EASD (45) and Turkish Society of Endocrinology and Metabolism (TEMD) (63) guidelines, IDegAsp can be initiated directly in any (insulin-naïve) patient who failed to achieve adequate glycemic control [HbA1c levels >2% of the individualized HbA1c target and/or HbA1c >8.5% despite optimal OADs (metformin, 2OADs, 3OADs) therapy in adults and HbA1c ≥7.5% despite metformin for T2D children], and who need a better post-prandial glycemic control. Adult patients with inadequately controlled blood glucose levels on OAD, IDegAsp may be more effective particularly in patients with HbA1c lower than 8.5.The recommended starting total daily dose of IDegAsp is 10 units with meal(s), whereas using 0.1–0.2 U/kg dose calculation may also be appropriate, and the individual dose adjustments should be performed at least after 48 h, as the time required for IDegAsp to reach steady state (29, 42, 43) (**Table 2**).

**Table 2:** Expert Panel Recommendation 2: Insulin-naïve patients.

• IDegAsp can be initiated directly in any (insulin-naïve) patient who have not achieved adequate glycemic control despite optimal OADs (metformin, 2OADs, 3OADs) • IDegAsp may be initiated as BID in case of unacceptable PPG is apparent at two mealtimes • HbA1c levels >2% of the individualized HbA1c target and/or • HbA1c >8.5% (HbA1c ≥7.5% despite metformin for T2D children) • Also in patients using basal insulin who need a better post-prandial glycemic control

##### Switching From Other Regimens in Insulin-Treated Patients

###### Switching From Basal Insulin

Switching to IDegAsp is considered a preferable alternative for treatment intensification in people with T2D with inadequate glycemic control or nocturnal hypoglycemia on basal insulin due to its low glucose-lowering variability (56).

In a 38-week, randomized, open-label, treat-to-target trial in adults T2D patients (on basal insulin ± OADs; HbA1c 7.0–10.0%) who were randomized (1:1) into IDegAsp or IGlar U100 + IAsp, the target HbA1c achievement, mean fasting and postprandial glucose levels and safety profile were reported to be similar across groups, while IDegAsp was associated with higher rate of achieving target HbA1c without hypoglycemia (22.5 vs. 21.2% at the end of 38 weeks), significant risk reduction for nocturnal hypoglycemia (by 45 and 39% at the end of 26 and 38 weeks, respectively) and 10.7% reduction in insulin need at 26 weeks and 6.6% lower dose at 38 weeks (56). Authors also noted once daily/twice daily (OD/BID) IDegAsp may be more effective treatment intensification options versus multiple injection basal–bolus therapies, achieving similar glycemic control, with significantly less nocturnal hypoglycemia and lower insulin doses (56).

Recently, Cho et al. evaluated 59 Japanese patients (aged 20–80 years) with T2D who were on basal insulin (insulin degludec or glargine) at least for 12 weeks before enrollment. A 12 week, multicenter, open-label, randomized, prospective, parallel-group comparison, treat to target study was conducted (64). Patients were randomly assigned to continue basal insulin (n = 29) or to switch to IDegAsp (n = 30). Both groups were similar in terms of body mass index, HbA1c level and the identity of basal insulin used. Initial dose of IDegAsp was same with the basal insulin dose; however, the dose was titrated at the discretion of the physician. Concomitant medications were not changed throughout the study (65). The primary endpoint was to see the superiority of IDeg/Asp over basal insulin in patients with T2D. At the end of the 12 weeks, HbA1c level was significantly decreased (7.5% to 7.3%) only in the IDegAsp group. IDegAsp was more effective in controlling blood glucose after dinner and before bedtime without causing any increase in hypoglycemia (64).

The expert panel recommends that switching from basal insulin to IDegAsp can be applied to all T2D patients who have not achieved an individualized HbA1c target as accompanied with high FPG or high post-prandial glucose (PPG) under optimally titrated basal insulin treatment. The lesser rate of nocturnal hypoglycemia with IDegAsp as compared with basal insulin, owing to long-term efficacy of basal component, seems to be the one of the most important indications for switching to IDegAsp. The switching should be performed 1:1 without dose reduction, while with dose titration based on FPG values as well as PPG values after the meal with injection, measured at least weekly (**Table 3**).

**Table 3:** Expert Panel Recommendation 3: Switching from basal insulin.

• Failure to achieve an individualized HbA1c target as accompanied with high FPG or high PPG under optimally titrated (3–6 months) basal insulin treatment • To obtain lesser rate of nocturnal hypoglycemia • The switching should be performed 1:1 without dose reduction • The dose titration should be based on FPG values as well as PPG values after the meal with injection, as measured at lebull weekly

###### Twice Daily IDegAsp Use

The expert panel recommends that twice daily IDegAsp can be used when daily insulin need exceeds 0.5 U/kg under OD IDegAsp treatment, by splitting the total daily dose (0.5U/kg) of IDegAsp OD into two doses, to be administered at the two largest meals by carbohydrate. The expert panel also agreed on the likelihood of switching directly to two-dose IDegAsp in patients with very poor glycemic control (HbA1c >9%) with high FPG levels (>250 mg/dL) or post-prandial glucose excursions after two meals and in patients with postprandial hypoglycemia under once daily IDegAsp treatment or when an increase in the basal insulin dose is needed, as an alternative to basal bolus regimen (**Table 4**).

**Table 4:** Expert Panel Recommendation 4: Twice daily IDegAsp use.

*In patients under single-dose IDegAsp therapy:* • when daily insulin need exceeds 0.5U/kg • total daily dose (0.5U/kg) of IDegAsp OD is split into two doses • the ratio of split (50:50 is not mandatory) should be based on the relative size of the meals, taking the carbohydrate content into consideration *In patients under basal insulin therapy:* • switching directly to two-dose IDegAsp as an alternative to basal bolus regimen • Poor glycemic control (HbA1c >9%) • Lack of FPG control (>250 mg/dL) • Lack of PPG control for more than one meal • Postprandial hypoglycemia under once daily IDegAsp treatment • A need for increase in the basal insulin dose

###### Switching From Basal-Bolus Regimen

IDegAsp is considered suitable for patients who do not want to take multiple injections each day, and may therefore provide an alternative to basal–bolus regimens (38). Notably, in a randomized trial with T2D patients, IDegAsp BID versus IDeg OD + IAsp 2–4 times was reported to be associated with improved patient-reported outcome scores for social functioning, while the two treatment groups were similar in terms of glycemic control or hypoglycemia (38). Authors also noted the likelihood of reduced burden of injections with IDegAsp versus IDeg +IAsp to result in improved patient-reported outcome scores (38).

IDegAsp (having an algorithm similar to the basal treatment algorithm, maintaining familiarity and simplicity for the patient, through a unit-to-unit transfer) is considered to be a more effective treatment intensification option than multiple injection basal–bolus therapies (being more complex due to requirements for more injections and use of two titration algorithms), achieving similar glycemic control with significantly less nocturnal hypoglycemia and lower insulin doses (56). Moreover, the further intensification of IDegAsp OD is possible by splitting the dose when necessary, which also is considered as effective for intensification of treatment as a multiple injection basal–bolus regimen along with a more favorable nocturnal hypoglycemia profile (56). Hence, IDegAsp offers a simple, adaptable treatment option for patients in need of intensification with no major safety concerns regarding the switch from previous regimens, along with likelihood of better suitability to patient lifestyle than more complex alternatives, and with a lower daily injection frequency and lower risk of nocturnal hypoglycemia (due to a very flat glucose-lowering PK/PD profile with low variability) compared with basal–bolus regimens (56).

Accordingly, the expert panel recommends that in patients with poor adherence with basal bolus therapy, if Asp ratio does not exceed 30% of the total dose, the total dose calculated for basal bolus insulin can be injected in two equal doses (2x1 IDegAsp), whereas when IAsp ratio is >30% of the total dose, switching to a 3-dose therapy (IDegAsp + IAsp + IAsp) is more appropriate (**Table 5**).

**Table 5:** Expert Panel Recommendation 5: Switching from basal-bolus regimen.

*From 4 injection basal bolus therapy to 3 injections (IDegAsp + IAsp + IAsp):* • If Asp ratio is >30% of the total dose • Based on maintenance of the same basal dose • Based on 10–20% dose reduction in total dose and giving 50% as IDegAsp • Based on bolus dose if basal ratio >40–45%, while based on basal dose only if basal ratio is lower (Tip 1 diabetics) *From 4 injection basal bolus therapy to 2 injections (2X1 IDegAsp)* • If Asp ratio does not exceed 30% of the total dose • Owing to similar efficacy of IDegAsp with two injections • With individualized treatment doses being titrated to the FPG and PPG Note: In patients receiving basal-bolus treatment, particularly those need high daily insulin doses (i.e., >100 U), achievement of glycemic control via 2X1 IDegAsp is challenging and these two approaches should not be considered as appropriate alternatives of each other in every case. In switching from basal-bolus regimen to IDegAsp, presence of mandatory conditions or obvious lack of compliance is important to be considered in treatment decision.

###### Switching From Four Injection Basal Bolus Therapy to Three Injections (IDegAsp + IAsp + IAsp)

This is appropriate in T2D and T1D patients with high number of daily insulin injections (to enable lesser number of injections), with poor treatment adherence (to improve adherence), with nocturnal hypoglycemia and with an increase in the basal insulin dose need. The experts also agreed that while switching from basal bolus to IDegAsp is recommended to be based on maintenance of the same basal dose and this has been probably associated with lesser postprandial insulin need after switching, the dose adjustment according to Asp dose seems also be rational to prevent hypoglycemia, given the more stable glycemic control with IDegAsp even at lower doses (**Table 5**).

The expert panel recommends that in T1D patients, switching according to bolus-based calculation leads to overtreatment in patients with low basal ratio in terms of IDegAsp (long-insulin), necessitating 10–20% dose reduction in total dose to prevent hypoglycemia. Accordingly, only if basal ratio >40–45%, the dose calculation should be based on bolus dose and IDegAsp should comprise 50% of the calculated dose (**Table 5**).

###### Switching From Four Injection Basal Bolus Therapy to Two Injections (2X1 IDegAsp)

The expert panel recommends that in T2D patients with very high basal insulin need, poor treatment adherence switching to two dose IDegAsp can be performed (via dividing total dose into two) on the basis of Asp (short insulin) need. Since 2x1 IDegAsp offers similar efficacy with basal bolus therapy, switching from four-injection basal bolus to two-injection IDegAsp may be an alternative in patients with poor adherence with basal bolus therapy, in accordance with individualized treatment doses being titrated to the FPG and PPG (**Table 5**).

###### Switching From Premixed Insulins

In a 26-week, randomized, open-label, multinational, treat-to-target trial comparing BID injections of IDegAsp (n =224) or BIAsp 30 (n = 222) in patients with uncontrolled T2D previously treated with once- or twice-daily pre- or self-mixed insulin, IDegAsp BID was reported to be associated with similar/non-inferior HbA1c reductions and superior FPG reductions with fewer hypoglycemia episodes versus BIAsp 30 (35).

In a study among adult T2D patients randomized 2:1 to BID IDegAsp (n = 282) or BIAsp 30 (n = 142), IDegAsp was reported to be non-inferior to BIAsp 30 for mean change in HbA1c (ETD IDegAsp-BIAsp 30: 0.05% points [95% CI -0.10; 0.20], p = NS), while showed superiority for lowering FPG levels (ETD: -1.06 mmol/L, 95% CI -1.43; -0.70, p<0.001), mean daily insulin dose (0.79 U/kg vs 0.99 U/kg, estimated RR: 0.79, 95% CI 0.73; 0.85, p<0.0001) and numerically lower nocturnal hypoglycemia rate (1.1 vs. 1.6 episodes/patient/year; RR: 0.67, 95% CI 0.43; 1.06) than BIAsp 30 (36). Authors considered IDegAsp BID to effectively improve long-term glycemic control, and compared to BIAsp 30, to provide superior reductions in FPG with a lower dose, and numerically less nocturnal hypoglycemia (36).

In another study, combined analysis of two Phase 3a studies in type 2 diabetes patients treated twice daily with IDegAsp or BIAsp 30 over 26 weeks revealed that the rates of overall confirmed, nocturnal confirmed and severe hypoglycemic events were 19%, 57%, and 39% lower, respectively, with IDegAsp (n = 504) than BIAsp 30 (n = 364) (65). Authors indicated association of IDegAsp twice daily vs. BIAsp 30 twice daily treatment with similar improvements in glycemic control, whereas a lower risk of hypoglycemia, particularly nocturnal hypoglycemia, in T2D patients previously treated with insulin (65).

Accordingly, the expert panel recommends that in patients with overall or nocturnal hypoglycemia or a need for dose reduction, switching from premix insulins can be performed with 1:1 dose ratio in patients with poor glycemic control, while with 10 % dose reduction in patients who are at target and metabolically controlled (**Table 6**).

**Table 6:** Expert Panel Recommendation 6: Switching from premixed insulins.

• With 1:1 dose ratio in poor glycemic control • With 10 % dose reduction in patients who are at target HbA1c • In patients with nocturnal hypoglycemia or need for dose reduction	

##### Co-Administration With Other Antidiabetic Medications

The expert panel recommends that IDegAsp can be used in combination with most OADs and no additional considerations are required when combining IDegAsp with metformin or dipeptidyl peptidase 4 (DPP-4) inhibitors, which can each be continued at the same dose when IDegAsp is added (29). A reduction in insulin dose is necessary if sodium–glucose cotransporter 2 (SGLT-2) inhibitors are added to IDegAsp, and caution should also be taken when IDegAsp is used together with sulphonylureas (SUs) (29).

#### IDegAsp Therapy in Children With T2D

The incidence of T2D in adolescents has increased globally in recent decades, considered to be linked to obesity, while the rapid decline of β-cell function in adolescents merits the use of insulin treatment (66–69). Indeed, initial treatment with metformin and/or insulin alone or in combination is recommended in adolescents with T2D and marked hyperglycemic (FPG ≥250 mg/dL and/or HbA_1c_ ≥8.5%) (70).

Although IDegAsp has not been the subject of clinical trials in adolescents with T2D, analysis of efficacy and safety using data from adolescent and adult patients with T1D and adults with T2D, supports the use of IDegAsp in adolescent patients with T2D (29).

Accordingly, the expert panel recommends that in T2D children who need treatment intensification on metformin, first basal insulin is initiated and then IDegAsp can be used instead of basal bolus therapy, similar to adult age group, while in those with diabetic ketoacidosis intensive treatment is applied directly and IDegAsp may be administered after the remission **(** **Table 7** **).**

**Table 7:** Expert Panel Recommendation 7: Use in Children with type 2 DM.

• Similar to adult T2D patients except for insulin-naïve patients • As a basal bolus alternative rather than as an alternative to basal insulin after metformin failure

### Use In Pediatric and Adult T1D Patient Populations

#### IDegAsp Therapy in Adult Patients With T1D

IDegAsp is mainly used as a treatment for T2D and is not commonly used to treat patients with T1D. However, in adults with T1D, IDegAsp as part of a simplified basal-bolus regimen with IAsp was observed to improve overall glycemic control and was non-inferior to IDet + IAsp basal–bolus therapy (HbA1c improved by 0.75% with IDegAsp and 0.70% with IDet to 7.6% in both groups) as well as incurring a relatively reduced risk of nocturnal hypoglycemia (3.71 vs. 5.72 episodes/patient-year, p < 0.05) (31).

In a past study with T1D patients randomized to IDegAsp once daily at the main meal and IAsp at remaining meals (IDegAsp+IAsp), or IDet (once or twice daily) and IAsp at all meals (IDet+IAsp), once-daily treatment with IDegAsp and IAsp as bolus insulin for remaining meals was reported to be associated with significantly lower risk of nocturnal confirmed hypoglycemia (3.1 vs. 5.4 episodes/patient-year exposure, respectively; p < 0.05), improved glycemic control and showed non-inferiority compared with IDet+IAsp (mean HbA1c decrease from baseline by 0.7 and 0.6% at week 52, respectively), the standard of care in Type 1 diabetes (32). Authors considered the achievement of intensive insulin therapy with IDegAsp+IAsp with three injections instead of a minimum of four in T1D patients along with effective glycemic control over 52 weeks with one less injection compared with a conventional basal–bolus insulin regimen to be of real value to participants by alleviating the injection burden, and thereby potentially improving adherence and quality of life (32).

Accordingly, the expert panel recommends that in adult patients with T1D, IDegAsp +IAsp treatment can be used to simplify basal bolus treatment in terms of fewer injections, better FPG control, lesser rate of nocturnal hypoglycemia and reduction in insulin dose. The dose should be individualized (**Table 8**). If the patient is taking a fixed amount of carbohydrates, IDegAsp +IAsp is recommended. If the patient is counting carbohydrates, care should be taken in terms of hypoglycemia.

**Table 8:** Expert Panel Recommendation 8: Use in Type 1 DM.

*Adults* • Simplified basal bolus regimen • lesser nocturnal hypoglycemia, fewer injections and better FPG control *Children* • > 2-years of age with o insufficiency of long-acting insulin o skipping injections o diabetic ketoacidosis risk due to missed injections o fear of injection • two basal dose formulation: calculated based on bolus dose (not exceed the total basal dose) • one basal dose formulation: calculated based on bolus dose if basal ratio >40–45%, if basal ratio <30%.comprising 50–55% of the dose after 10–20% dose reduction

#### IDegAsp Therapy in Children With T1D

IDegAsp treatment could be preferable insulin regimen among children for its single injection pen when the injections are frequently missed by children. However, usually at least two additional meal time insulin boluses are required. There is no clinical experience with the use of IDegAsp in children aged <2 years and special caution should be taken for children aged 2–5 years, as clinical data suggest a higher risk of hypoglycemia in this age compared with children aged 5–17 years (29).

In a 16-week, phase 3b, treat-to-target, parallel-group, open-label, non-inferiority trial in children and adolescents with T1D randomized 1:1 to IDegAsp OD plus IAsp for remaining meals (IDegAsp + IAsp, n = 182), or IDet OD or twice daily plus mealtime IAsp (IDet + IAsp, n = 180), authors reported IDegAsp + IAsp to provide similar glycemic control compared with IDet + IAsp (HbA1c decreased from baseline to week 16 by 0.3% in both groups), with the benefit of fewer injections per day (mean 3.6 and 4.9) (71). Authors indicated IDegAsp + IAsp was non-inferior to IDet + IAsp regarding HbA1c, had similar hypoglycemia rates and required fewer injections (71).

Accordingly, the expert panel recommends that IDegAsp can be recommended in T1D children (>2-years of age) with the number of injections increased due to insufficiency of long-acting insulin, in those who frequently skip injections with subsequent risk of diabetic ketoacidosis, those with fear of injection, and in those need alternative insulin regimens due to inability to use insulin pump. For patients taking two-basal dose, IDegAsp dose is calculated based on bolus dose (basal dose divided by 0.3), while calculated IDegAsp dose should not exceed the total basal dose. For switching from single basal injection (i.e. for morning hypoglycemia or need for an increase in dose), the dose also can be calculated based on bolus dose, if basal ratio >40–45%. While basal ratio is low (<30%), 50% of total daily dose could be given as IDegAsp dose after 10–20% dose reduction of total daily (**Table 8**).

### Use in Pregnancy

The short acting component of insulin IDegAsp has been used for several years for the treatment of pregnant patients either with gestational diabetes or in women with T1D/T2D. However, the long acting component “degludec insulin” is not approved in pregnancy yet. IDeg is a category C agent in pregnancy. There are no published randomized controlled trials of IDeg in pregnant women and only case series have been reported. A review of six cases born from the mothers used IDeg during their pregnancy; none of the neonates showed malformation, however, three babies had respiratory distress, bilirubin increase or hypoglycemia (72). Hiranput et al. (73) also reported the effects of IDeg in three pregnancies who were not tolerated (having hypoglycemia) the approved basal insulins, and indicated healthy babies were born and the patients had improved glucose control during pregnancy However, more information is needed regarding the safety of IDeg in pregnancy. The ongoing trial “Research Study Comparing Insulin Degludec to Insulin Detemir Together with Insulin Aspart, in Pregnant Women with Type 1 Diabetes” (EXCPECT study-NCT03377699) is aimed to compare the effectiveness and the safety of insulin degludec in comparison to insulin detemir together with insulin aspart in pregnant women with type 1 diabetes mellitus. Until more data obtained, the expert panel recommends against using IDegAsp in pregnant women (**Table 9**).

**Table 9:** Expert Panel Recommendation 9: Use in Pregnant Patients.

• ‘Not recommended because of IDeg component of the co-formulation	

### Use in Special Patient Groups

#### Hospitalized Patients

Rapid-acting insulin is generally preferred to combination insulins in hospitalized patients, due to greater flexibility in titrating. IDegAsp may not be practical for titrating doses for in-patients given the time taken for the IDeg component to reach steady state, and the fixed ratio of the IAsp content. This may be pertinent when there are changes to diet, appetite, cases of sepsis and the need to take corticosteroids. In such cases, an alternate insulin regimen may be more appropriate (e.g., basal–bolus insulin or premix insulin).

Accordingly, the expert panel recommends that for major operations, IDegAsp treatment needs to be discontinued 24 h before the operation. For elective minor interventions (i.e. colonoscopy), the insulin dose may need to be decreased ≈3 days prior to the procedure or patients may be switched from IDegAsp to BIAsp 30 BID or basal insulin or basal-plus-bolus insulin. In patients hospitalized for evaluation purposes basal plus-bolus treatment may be a good alternative or, treatment can be continued with IDegAsp in those without injection or poor oral intake (**Table 10**).

**Table 10:** Expert Panel Recommendation 10: Use in Special Patient Groups.

Hospitalized patients: • For major operations, IDegAsp treatment needs to be discontinued 24 hours before the operation. • For elective minor interventions (i.e., colonoscopy), the insulin dose may need to be decreased ≈3 days prior to the procedure or patients may be switched from IDegAsp to BIAsp 30 BID or basal insulin or basal plus-bolus insulin • In patients hospitalized for evaluation purposes basal plus-bolus treatment may be a good alternative or, treatment can be continued in those without injection or poor oral intake Elderly patients: • Increased risk of hypoglycemia and need for less stringent HbA1c targets • Flexibility in dose timing may be advantageous for elderly patients in nursing homes • safe in elderly Renal or hepatic impairment • Not affected, while careful dose titrations are important for hypoglycemia risk IDegAsp is not a good alternative for patients with steroid induced/associated hyperglycemia particularly when steroid doses are rapidly changing.

#### Elderly Patients

In elderly patients, it is important to consider the increased risk of hypoglycemia, particularly debilitating due to frailty as well as less stringent HbA_1c_ targets (<8% or ≤9%) than younger patients (74, 75) (**Table 10**).

In a recent study in elderly (aged ≥65 years) T2D patients, IDegAsp treatment was reported to provide effective glycemic control consistent with the effects of BIAsp 30 (ETD IDegAsp–BIAsp 30: −0.02% [− 0.19; 0.15] 95% CI, p = 0.8455) and similar overall confirmed (estimated RR: 0.92 [0.67; 1.26] 95% CI, p = 0.5980) or nocturnal (estimated RR IDegAsp/BIAsp 30: 0.67 [0.39; 1.18] 95% CI, p = 0.1676) hypoglycemic events (76). In addition, the glucose-lowering effects of basal and prandial components of IDegAsp are considered to be maintained in elderly (≥65 years of age) T1D patients (43, 77).

The flexibility in dose timing of IDegAsp may be advantageous in elderly patients being treated in nursing homes or at home by visiting nurses given the likelihood of delay in care, while SUs should be discontinued when IDegAsp treatment is started, owing to the increased risk of hypoglycemia (42).

#### Other Patient Groups

In addition, the PK and clearance of IDeg and IAsp are considered not affected by mild, moderate, or severe renal or hepatic impairment (23, 43), same principles with insulin therapy apply, while due to hypoglycemia risk dose titrations should be performed carefully (**Table 10**).

## Conclusions

It is clear that there is no any ideal treatment for the management of diabetes mellitus. Optimal treatment for a given patient should be arranged according to his/her life style, co-morbidities etc. and his/her management may be changed in some special conditions. This expert panel statement provide recommendations for use of IDegAsp in clinical practice for different patient populations (T1D, T2D, children, adults, pregnant, elderly, hospitalized patients) and for varying practice patterns (insulin-naive, insulin-treated, switching from basal, basal bolus and premix regimens) to assist clinicians in using this novel insulin regimen in clinical practice.

Accordingly, the expert panel emphasized the superior PK profile of IDegAsp to premix insulins, due to availability of insulin components as separate and stable soluble forms in the formulation, thereby avoiding the need for resuspension prior to injection as well as distinct and clearly separated prandial and basal glucose-lowering effects at steady state. The experts consider that IDegAsp provides basal as well as prandial insulin cover in a single injection, while the co-formulation provides dosing flexibility and thus fewer injections as compared with basal–bolus regimens.

The experts consider the main meal, the most carbohydrate-rich meal, to be at any time during the day or at different times from day to day owing to flexibility in dose timing of IDegAsp, while they also emphasized that the main meal can be accurately identified based on analysis of self-measured blood glucose levels.

The expert panel consider the use of IDegAsp in insulin-naïve T2D patients with poor glycemic control (HbA1c >8.5%) despite optimal OADs as well as in insulin-treated T2D patients by switching from basal insulin, basal-bolus therapy or premixed insulins in relation to lower risk of nocturnal hypoglycemia, fewer injections and lower intraday glycemic variability, respectively. The experts consider the use of IDegAsp in children with T2D as a basal bolus alternative rather than as an alternative to basal insulin after metformin failure, use of IDegAsp in adult T1D patients as a simplified basal bolus regimen with lesser nocturnal hypoglycemia, fewer injections and better FPG control and in children with T1D as an alternative insulin regimen with fewer injections to increase treatment adherence. Until more data obtained, the expert panel recommends against using IDegAsp in pregnant women. Considering the limitations of co-formulation, although it may suffice for many patients with T2D, IDegAsp is not an ideal option for patients with possible inadequate insulin reserve. IDegAsp co-formulation is less flexible treatment option in comparison to basal-plus therapy. Intention to increase basal or bolus component only is not possible in co-formulation insulin therapies. Accordingly, this expert panel-based consensus statement provides practical national guidance on use of IDegAsp in different patient populations and practice patterns to assist clinicians, which seems to compensate the need for easily implementable guidelines on this novel insulin regimen. Further studies including observational studies comparing IDegAsp with other insulin regimens (premix, basal, basal-bolus) in terms of HbA1c reduction and rates of hypoglycemia and patient-reported outcome questionnaires, which assess the quality of life and adherence, together with physician surveys that measure provider experience, are required to evaluate the impact of this co-formulation on routine clinical practice.

## Author Contributions

All authors contributed equally to work. DG had primary responsibility for the manuscript preparation. All authors contributed to the article and approved the submitted version.

## Funding

This expert panel study was supported by Novo Nordisk Turkey, which played a role in organization of expert panel meetings including invitation of participants and compensation for the time and transport expenses of the experts. Editorial support was provided by KAPPA Consultancy Training Research Ltd., Istanbul, Turkey (supported by Novo Nordisk). All authors contributed to the discussion and preparation of the expert opinion and critically revised and approved the manuscript, and the authors take full responsibility for the contents of the article.

## Conflict of Interest

The authors declare that the research was conducted in the absence of any commercial or financial relationships that could be construed as a potential conflict of interest.
